# Supplementary material for: Limit state equation and failure pressure prediction model of pipeline with complex loading
Source: Nat Commun. 2024 May 25;15:4473. doi: 10.1038/s41467-024-48688-1 (PMC11127988; doi:10.1038/s41467-024-48688-1)
Supplement: Supplementary file 1 — Supplementary Information [file 41467_2024_48688_MOESM1_ESM.pdf]

Supplementary information
Research on the Limit State Equation and Failure Pressure Prediction Model of Pipeline with Complex Loading

SUN Ming-ming[1,2,3], FANG Hong-yuan[1,2,3*], WANG Nian-nian[1,2,3], DU Xue-ming[1,2,3], ZHAO Hai-sheng[4,5], ZHAI Ke-Jie[1,2,3]

1. School of Water Conservancy and Transportation, Zhengzhou University, Zhengzhou 450001, China; 2. National Local Joint Engineering Laboratory of Major Infrastructure Testing and Rehabilitation Technology, Zhengzhou 450001, China; 3. Collaborative Innovation Center of Water Conservancy and Transportation Infrastructure Safety, Zhengzhou 450001, China; 4. State Key Laboratory of Coastal and Offshore Engineering, Dalian University of Technology, Dalian 116024, China; 5. School of Hydraulic Engineering, Faculty of Infrastructure Engineering, Dalian University of Technology, Dalian 116024, China

Contents

Supplementary Figure 1 to 5

Supplementary Note 1

Supplementary Table 1

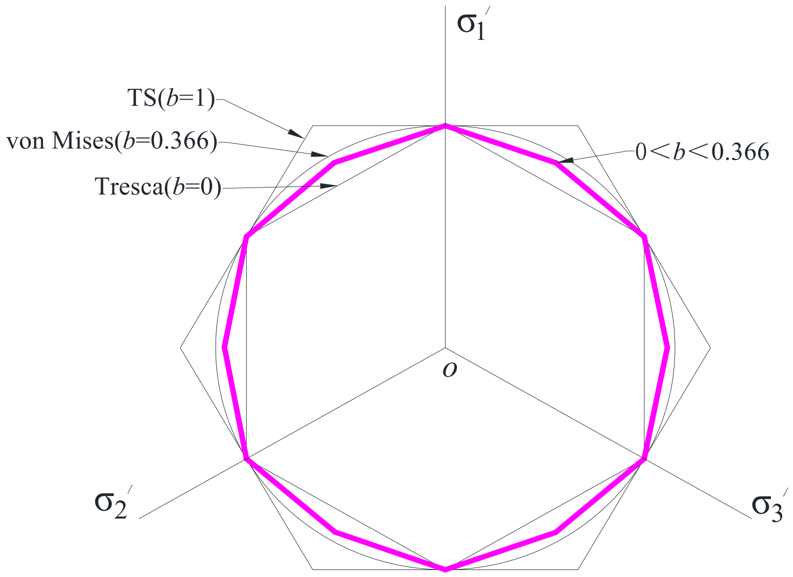

Supplementary Fig. 1. Yield criteria on the π-plane. The yield surfaces of different yield criteria are projected onto the π plane. The TS and Tresca criteria are projected as regular hexagons, the von Mises criteria are projected as circles, and the other criteria are projected as regular twelve deformations. The TS criterion has the largest range of yield trajectories and the Tresca criterion has the smallest range.

(a) (b)

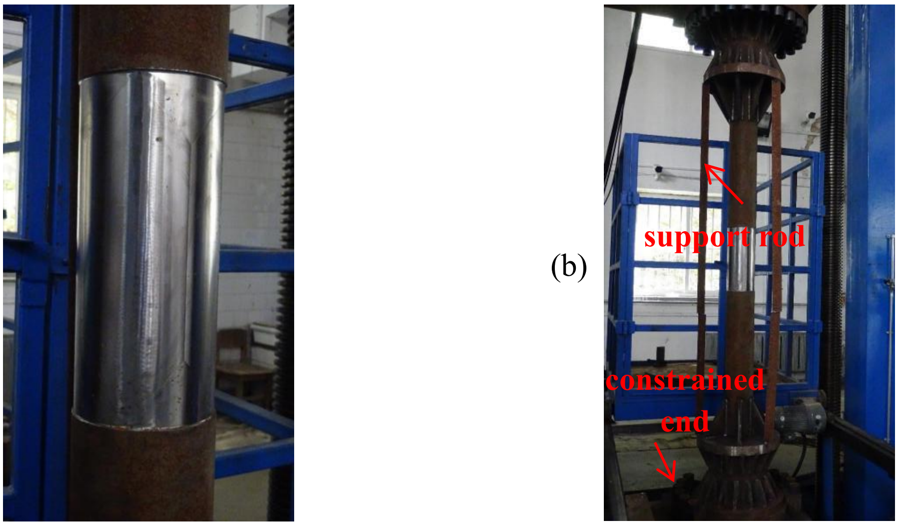

Supplementary Fig. 2| Q235 # 1 The test Pipeline. **a** Partial thinning section of the pipeline. **b** Overall diagram of the test pipeline. The total length of the test pipeline is L_0=2800mm, and the end is fixed and sealed by welding with the flange plate. Support rod was used to prevent damage to the reduction section during transportation and was removed in the experiment. The flange plate is reserved with inlet and outlet holes, as shown in **Supplementary Fig. 3**. Thinned pipeline section is located in the middle of the pipeline, and the distance between each flange plate and the edge of the nearest thinned section is greater than 2.4D_e, which is sufficient to avoid any form of interaction between the defect and the flange plate.

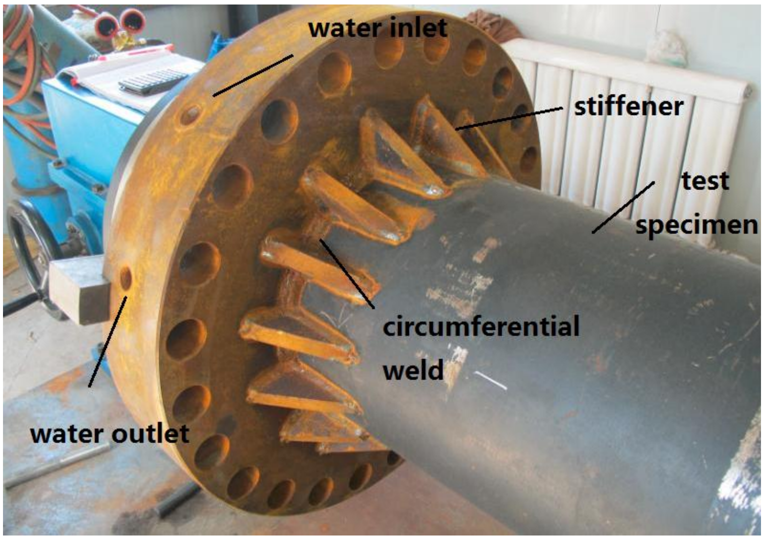

Supplementary Fig. 3| Sealing processing of test pipeline. The closure and end fixation of the test is shown in the figure. The end of the pipeline is closed with a flange plate, and there is a 20mm groove inside the flange plate to allow the pipeline to be inserted and provide better sealing effect. Circumferential welding shall be carried out at the connection between the pipeline and the flange plate. To enhance the connection between the pipeline and the flange plate, a stiffener is welded to the pipeline. There are water injection holes and outlet holes on the side of the flange, which are connected to the water supply pipe through joints and fixed and sealed with nuts. The flange plate has bolt holes, which are linked to the loading device through bolts for axial force loading.

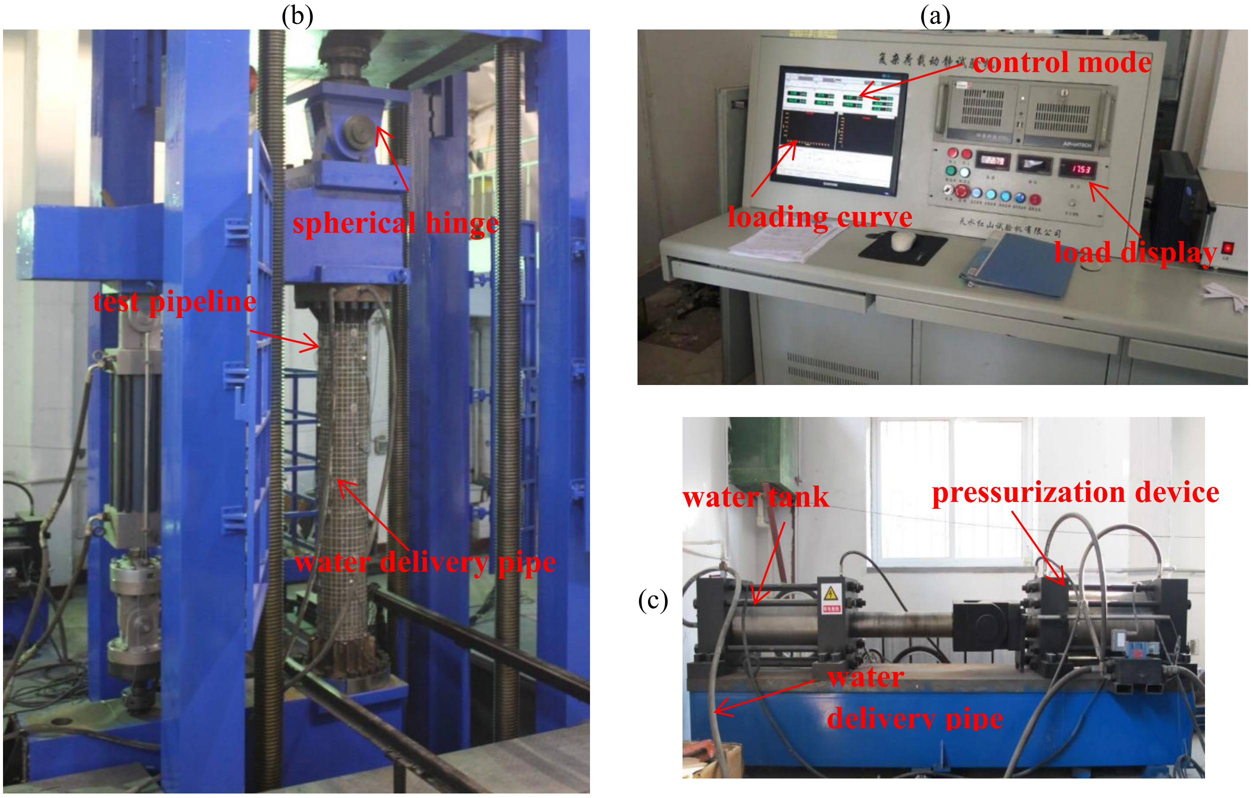

Supplementary Fig. 4| Loading device and control platform for pipeline tests. **a** Control platform. The control platform can control the pressure application method through sensors, including displacement control and load (pressure) control. **b** Axial force loading system. The 6000 kN hydraulics cylinder is installed inside the pipeline base and can apply an axial force of -6000 kN (in compression)~2000 kN (in tension). **c** The internal pressure loading device includes water injection, drainage, and pressurization devices. The pressurization device is a set of stainless steel boosting cylinders driven by servo oil cylinders, with a stroke of 600 mm and the ability to apply a maximum pressure of 50 MPa.

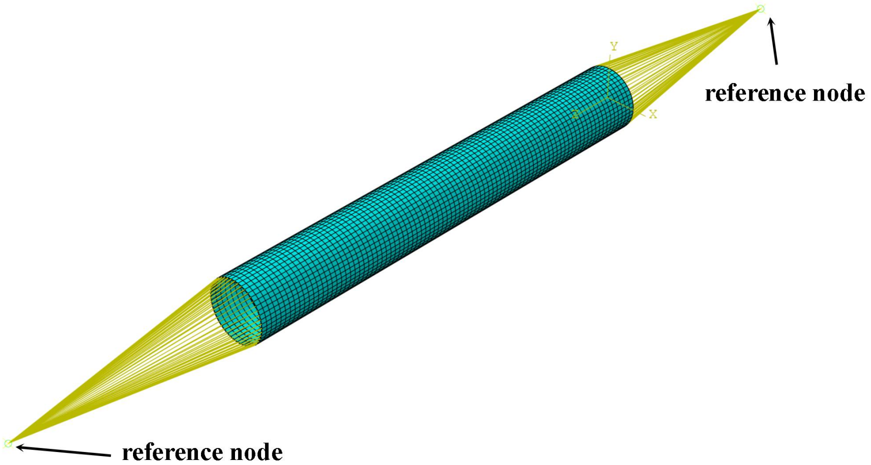

Supplementary Fig. 5. FE model for predicting the burst pressure. A finite element model of the pipeline was constructed using a three-dimensional solid unit C3D8R (as shown in blue in Supplementary Fig. 2). Use rigid beam constraints between the reference point and the end node of the pipeline (as shown in yellow in Supplementary Fig. 2). Apply bending moment and axial force on the reference point, and apply internal pressure load on the inner surface nodes of the pipeline model.

Supplementary Note 1

The process of loading. The pipeline burst test with internal pressure and axial force was conducted by applying a load through oil pressure, and the specific experimental setup is shown in Supplementary Fig. 4. The experimental steps are as follows:

(1) Connect the water injection and drainage pipes to the test pipeline and inject water into the test pipeline through tap water. After the drainage pipe overflows for a period of time, close the outlet pipe and inlet valve to ensure that the test pipeline and booster cylinder are fully vented and filled with water. The water injection of procedure (1) mainly fills the internal space of the pipeline through water injection and discharges air.

(2) At the beginning of the test, the axial force was loaded to design value with force control and remained unchanged.

(3) The pressurization device applies pressure to the pipeline through the movement of the piston in the water tank.

(4) Force controlled loading is adopted and the pressure increasing rate was 0.25 MPa/min. As most measuring points enter the yield stage, switch to displacement control. The rate of water injection volume was maintained at a limit of 0.3 cm^3/min until the pipeline fails due to burst.

Supplementary Table 1. Comparison of predicted values for different yield criteria (burst test data)

**case**	failure pressure (MPa)	predicted value of different yield criterias (MPa)				error analysis of different yield criterias			
		Tresca (*b*=0)	ASSY (*b*=0.168)	von Mises (*b*=0.366)	TS (*b*=1)	Tresca (*b*=0)	ASSY (*b*=0.168)	von Mises (*b*=0.366)	TS (*b*=1)
test in the paper	11.69	11.38	11.97	12.57	13.94	-2.66%	2.42%	7.50%	19.25%
13	115.5	120	117.91	116.47	114.44	3.91%	2.10%	0.86%	-0.90%
16	64.1	39.32	55.16	60.18	72.23	-38.64%	-13.92%	-6.08%	12.72%
17	50.6	26.28	41.19	51.36	65.86	-48.11%	-18.67%	1.43%	30.06%
18	54.8	44.03	56.34	49.67	52.92	-19.71%	2.73%	-9.43%	-3.50%
19	63.4	25.45	48.24	63.81	85.96	-59.85%	-23.90%	0.66%	35.60%
22℃-1	63	66.44	67.23	68.01	69.83	5.46%	6.71%	7.96%	10.85%
22℃-2	60	66.44	67.23	68.01	69.83	10.73%	12.04%	13.36%	16.39%
22℃-3	58	66.44	67.13	67.82	69.42	14.55%	15.74%	16.93%	19.68%
22℃-4	65	64.13	64.71	65.10	65.67	-1.35%	-0.45%	0.16%	1.03%
22℃-5	63	65.38	65.64	65.82	66.08	3.77%	4.19%	4.48%	4.89%
22℃-6	63	66.44	65.29	64.15	61.50	5.46%	3.64%	1.82%	-2.38%
22℃-7	59	66.44	54.88	57.52	61.29	12.61%	-6.99%	-2.50%	3.88%
22℃-8	54	49.75	53.94	56.80	60.88	-7.87%	-0.11%	5.19%	12.73%
22℃-9	61.5	66.44	64.33	62.21	57.33	8.03%	4.59%	1.16%	-6.78%
22℃-10	59	66.44	64.32	62.19	57.29	12.61%	9.01%	5.41%	-2.90%
22℃-11	57	66.44	64.42	62.41	57.75	16.56%	13.02%	9.48%	1.32%
22℃-12	54.5	66.44	45.98	50.67	57.33	21.90%	-15.63%	-7.03%	5.20%

90℃-1	62.5	59.25	59.97	60.68	62.33	-5.20%	-4.06%	-2.91%	-0.27%
90℃-2	55	59.25	59.87	60.49	61.92	7.73%	8.85%	9.98%	12.58%
90℃-3	54	59.25	59.87	60.49	61.92	9.72%	10.87%	12.01%	14.66%
90℃-4	55	59.25	52.14	53.77	56.08	7.73%	-5.21%	-2.25%	1.97%
90℃-5	54.5	59.25	52.04	53.69	56.04	8.72%	-4.51%	-1.48%	2.83%
90℃-6	50.5	59.25	57.55	46.55	51.92	17.33%	13.96%	-7.82%	2.81%
110℃-1	65	58.44	59.09	63.31	61.25	-10.10%	-9.09%	-2.60%	-5.77%
110℃-2	61	58.44	59.09	59.74	61.25	-4.20%	-3.13%	-2.06%	0.41%
110℃-3	58.5	58.44	59.09	59.74	61.25	-0.11%	1.01%	2.12%	4.70%
110℃-4	56.5	58.44	57.64	56.84	55.00	3.43%	2.02%	0.61%	-2.65%
110℃-5	55	58.44	57.69	56.94	55.21	6.25%	4.89%	3.53%	0.38%
110℃-6	53.4	58.44	50.71	52.48	55.00	9.43%	-5.03%	-1.72%	3.00%
160℃-1	53	55.50	55.93	56.35	57.33	4.72%	5.52%	6.32%	8.18%
160℃-2	50.5	55.50	55.93	56.35	57.33	9.90%	10.74%	11.59%	13.53%
160℃-3	51.5	55.50	54.48	53.45	51.08	7.77%	5.78%	3.79%	-0.81%
160℃-4	48.5	42.25	45.58	47.85	51.08	-12.89%	-6.03%	-1.34%	5.33%
160℃-5	45.5	55.50	53.51	40.63	46.92	21.98%	17.60%	-10.70%	3.11%

Note:error= $\left(p_f - p_T\right)/p_T \times 100\%$. P_f is the predicted value, P_T is the failure pressure of test.
